# Real-World Dalbavancin Use for Serious Gram-Positive Infections: Comparing Outcomes Between People Who Use and Do Not Use Drugs

**DOI:** 10.1093/ofid/ofae186

**Published:** 2024-04-05

**Authors:** Sarah Zambrano, Molly L Paras, Joji Suzuki, Jeffrey C Pearson, Brandon Dionne, Harry Schrager, Jason Mallada, Veronica Szpak, Katie Fairbank-Haynes, Marlene Kalter, Sara Prostko, Daniel A Solomon

**Affiliations:** Brigham and Women’s Hospital, Department of Medicine, Harvard Medical School, Boston, Massachusetts, USA; Massachusetts General Hospital, Department of Medicine, Harvard Medical School, Boston, Massachusetts, USA; Brigham and Women’s Hospital, Department of Medicine, Harvard Medical School, Boston, Massachusetts, USA; Brigham and Women’s Hospital, Department of Medicine, Harvard Medical School, Boston, Massachusetts, USA; Brigham and Women’s Hospital, Department of Medicine, Harvard Medical School, Boston, Massachusetts, USA; Newton Wellesley Hospital, Department of Medicine, Boston, Massachusetts, USA; Newton Wellesley Hospital, Department of Medicine, Boston, Massachusetts, USA; Brigham and Women’s Hospital, Department of Medicine, Harvard Medical School, Boston, Massachusetts, USA; Newton Wellesley Hospital, Department of Medicine, Boston, Massachusetts, USA; Newton Wellesley Hospital, Department of Medicine, Boston, Massachusetts, USA; Brigham and Women’s Hospital, Department of Medicine, Harvard Medical School, Boston, Massachusetts, USA; Brigham and Women’s Hospital, Department of Medicine, Harvard Medical School, Boston, Massachusetts, USA

**Keywords:** bacteremia, dalbavancin, endocarditis, osteomyelitis, people who use drugs

## Abstract

**Background:**

Dalbavancin has been used off-label to treat invasive bacterial infections in vulnerable populations like people who use drugs (PWUD) because of its broad gram-positive coverage and unique pharmacological properties. This retrospective, multisite study examined clinical outcomes at 90 days in PWUD versus non-PWUD after secondary treatment with dalbavancin for bacteremia, endocarditis, osteomyelitis, septic arthritis, and epidural abscesses.

**Methods:**

Patients at 3 teaching hospitals who received dalbavancin for an invasive infection between March 2016 and May 2022 were included. Characteristics of PWUD and non-PWUD, infection highlights, hospital stay and treatment, and outcomes were compared using χ^2^ for categorical variables, *t* test for continuous variables, and nonparametric tests where appropriate.

**Results:**

There were a total of 176 patients; 78 were PWUD and 98 were non-PWUD. PWUD were more likely to have a patient-directed discharge (26.9% vs 3.1%; *P* < .001) and be lost to follow-up (20.5% vs 7.14%; *P* < .01). Assuming loss to follow-up did not achieve clinical cure, 73.1% of PWUD and 74.5% of non-PWUD achieved clinical cure at 90 days (*P* = .08).

**Conclusions:**

Dalbavancin was an effective treatment option for invasive gram-positive infections in our patient population. Despite higher rates of patient-directed discharge and loss to follow-up, PWUD had similar rates of clinical cure at 90 days compared to non-PWUD.

Invasive gram-positive infections including bacteremia, endocarditis, osteomyelitis, septic arthritis, and epidural abscesses are serious infections that can have devastating consequences [1, 2]. Standard of care for treatment of these infections typically entails 4- to 6-week courses of intravenous (IV) antibiotics, often requiring extended inpatient hospital stays and durable long-term IV access [3]. Prolonged treatment courses are particularly challenging for people who use drugs (PWUD), who often face structural barriers to care and numerous inequities within the healthcare system and who may be mistrustful of a healthcare system that has not prioritized their needs [4–6]. Additionally, PWUD admitted with severe injection-related infections have a high rate of patient-directed discharge (PDD) and loss to follow-up [7]. There is a growing body of literature supporting the use of oral antibiotics for severe infections, but PWUD are underrepresented in these data, and some patients face structural and medical barriers to adherence [8]. Therefore, alternative antibiotic strategies are needed to improve outcomes when treating severe infections in this population.

Dalbavancin, a long-acting lipoglycopeptide with a broad gram-positive spectrum of activity including methicillin-resistant *Staphylococcus aureus* (MRSA), is currently approved for skin and soft tissue infections but has also been used off-label to treat invasive gram-positive infections, including in PWUD [9–17]. Dalbavancin's key advantage is that it can be dosed once, weekly, or biweekly, eliminating the need for prolonged hospital stays and long_­_-term IV access. Several retrospective case series have described outcomes after use of dalbavancin in PWUD for a wide range of gram-positive infections including bacteremia, endocarditis, osteomyelitis, and septic arthritis and have found that dalbavancin was effective 56%–77% of the time [14–17]. However, these studies included only a small number of PWUD and many participants were lost to follow-up, so clinical outcomes data are limited. In this retrospective study, we reviewed the clinical outcomes of all patients with invasive gram-positive infections treated with off-label dalbavancin across 3 teaching hospitals and compared outcomes among PWUD to outcomes among non-PWUD.

## METHODS

### Study Design

This was a retrospective multisite observational study conducted at 3 university-affiliated hospitals in the Boston metropolitan area between March 2016 and May 2022. Patients were identified using pharmacy dalbavancin administration records during this time period. Patients included in the study were at least 18 years old, hospitalized with a serious infection (ie, isolated bacteremia, endocarditis, vertebral and nonvertebral osteomyelitis, septic arthritis, and epidural abscess), initially treated with an antibiotic other than dalbavancin, and received their first dose of dalbavancin during hospital admission. Patients who received dalbavancin for an isolated skin and soft tissue infection and patients who received their entire course of dalbavancin as an outpatient were excluded. This study received approval and exemption status from the institutional review board of the hospital system. Demographic data, past medical history, microbiologic data, and details of the hospital admission were collected directly from the medical record. Data were extracted from patient medical records and collected in Research Electronic Data Capture (REDcap) software. All data were verified by at least 2 investigators.

### Outcomes

The primary outcome was clinical cure at 90 days from the first dalbavancin dose without the need for rescue antibiotic therapy or hospital readmission for index infection. Secondary outcomes were rates of loss to follow-up, PDD, and clinical cure at 90 days in patients with a PDD.

### Definitions

PWUD was defined as anyone with active or a history of using at least 1 nonmarijuana drug including both injection and noninjection drug use. Details regarding timing and route of drug use were extracted. Clinical cure was defined as documentation in the medical record of at least 1 of the following: resolution of clinical signs and symptoms of infection without relapse of infectious symptoms, radiographic or microbiologic evidence of cure, and/or treating provider opinion that the infection was cured. Clinical cure was considered irrespective of whether the intended course of dalbavancin was completed. A patient was considered as being lost to follow-up if within the medical record there were no data at 90 days or beyond to assess whether there was clinical cure at the time of data extraction in 2023. PDD was defined as any PDD since diagnosis of index infection. Experiencing homelessness was defined as documentation by a provider that the patient was living on the street or in a shelter directly prior to admission for the infection. A patient was defined as actively being treated for a solid organ/hematological malignancy if they had received any cancer treatment, including bone marrow transplant and radiation therapy, within 6 months preceding hospitalization. A patient was defined as being on immunosuppressive therapy not related to malignancy if they were taking the equivalent of prednisone 20 mg a day for >2 weeks or cyclophosphamide, methotrexate, azathioprine, mercaptopurine, cyclosporine, tacrolimus, sirolimus, everolimus, mycophenolate mofetil, or biologic immunosuppressants/immunomodulators within 6 months prior to hospital admission for an indication not related to malignancy.

### Statistical Analysis

Descriptive statistics were used to summarize the data. Characteristics of PWUD and non-PWUD, infection highlights, hospital stay and treatment, and outcomes were compared using χ^2^ for categorical variables, *t* test for continuous variables, and nonparametric tests where appropriate. For all analyses, α was set to .05.

## RESULTS

**Figure 1.**
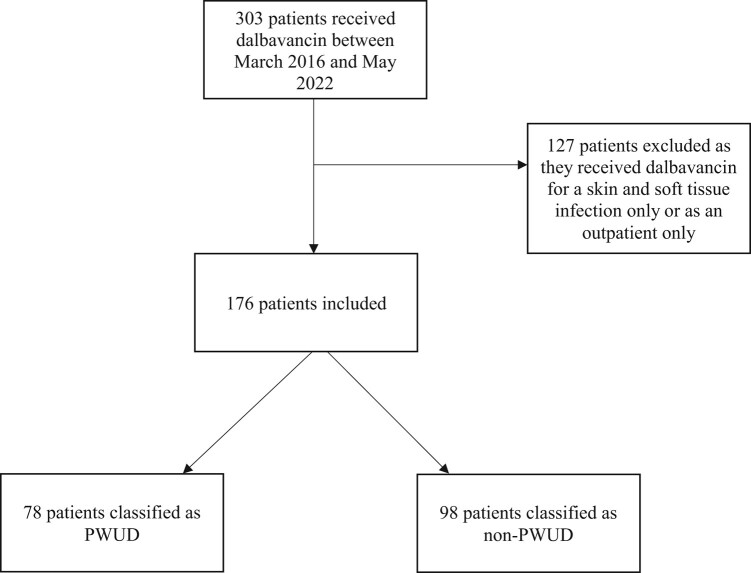
Study flowchart. Abbreviation: PWUD, people who use drugs.

A total of 303 patients were screened, with 176 patients meeting inclusion criteria as summarized in Figure 1. Of those, 78 were PWUD and 98 were non-PWUD. Demographic data are summarized in Table 1. There was no statistically significant difference in gender or race/ethnicity between groups. In both groups, nearly half of the patients identified as female and the majority identified as white non-Hispanic. PWUD were more likely to be younger (median age, 40.4 vs 56.2 years; *P* < .0001), to be experiencing homelessness (24.4% vs 2%; *P* < .0001), and to have a history of hepatitis C (65.4% vs 2%; *P* < .0001). Non-PWUD were more likely to have a history of diabetes mellitus (14.1% vs 32.7%; *P* < .01), to be on treatment for a solid or liquid malignancy within the last 6 months (5.1% vs 33.7%; *P* < .0001), and to be on non-malignancy-related immunosuppressive therapy within the last 6 months (1.3% vs 14.3%; *P* < .01). There was no statistical difference in prevalence of human immunodeficiency virus infection (7.7% vs 3.1%; *P* = .30) and glomerular filtration rate <30 mL/min at time of dalbavancin initiation (2.6% vs 11.2%; *P* = .05) between the groups. Among the PWUD group, 74.4% had a history of drug use within 6 months prior to admission and 47.4% reported IV drug use within 2 weeks prior to admission. Within the PWUD group, 41% were prescribed medication for opioid use disorder (MOUD) during the hospital admission.

**Table 1.** Patient Characteristics

Variable	Total (n = 176)	PWUD (n = 78)	Non-PWUD (n = 98)	*P* Value
Age, y, median (SD)	49.2 (17.0)	40.4 (11.1)	56.2 (17.7)	<.0001
Female gender	78 (44.3)	30 (38.5)	48 (49.0)	.22
White non-Hispanic	144 (81.8)	61(78.2)	83 (84.7)	.45
English language preference	165 (93.8)	76 (97.4)	89 (90.8)	.12
Experiencing homelessness	21 (11.9)	19 (24.4)	2 (2.0)	<.0001
History of diabetes mellitus	43 (24.4)	11 (14.1)	32 (32.7)	<.01
History of hepatitis C	53 (30.1)	51 (65.4)	2 (2.0)	<.0001
History of HIV	9 (5.1)	6 (7.7)	3 (3.1)	.30
Treated for malignancy^a^	37 (21.0)	4 (5.1)	33 (33.7)	<.0001
Treated with immunosuppressive agent^b^	15 (13.2)	1 (1.3)	14 (14.3)	<.01
GFR <30 mL/min at time of dalbavancin initiation	13 (7.4)	2 (2.6)	11 (11.2)	.05
MOUD during admission	32 (18.2)	32 (41)	0	<.0001
Any drug use <6** **mo prior to admission	58 (33.0)	58 (74.4)	0	<.0001
IV drug use <2** **wk prior to admission	37 (21.0)	37 (47.4)	0	<.0001

Data are presented as No. (%) unless otherwise indicated.

Abbreviations: GFR, glomerular filtration rate; HIV, human immunodeficiency virus; IV, intravenous; MOUD, medication for opioid use disorder; PWUD, people who use drugs; SD, standard deviation.

^a^Chemotherapy, radiation, or bone marrow transplant within the last 6 months.

^b^Prednisone 20 mg a day for >2 weeks or cyclophosphamide, methotrexate, azathioprine, mercaptopurine, cyclosporine, tacrolimus, sirolimus, everolimus, mycophenolate mofetil, or biologic immunosuppressants/immunomodulators within the last 6 months.

Infection information and microbiology is summarized in Table 2. Endocarditis (20.5% vs 5.1%; *P* < .01), epidural abscess (9% vs 1%; *P* < .05), and vertebral osteomyelitis (9% vs 0; *P* < .05) were more common in the PWUD group whereas isolated bacteremia (33.3% vs 57.1%; *P* < .01) and nonvertebral osteomyelitis (14.1% vs 27.6%; *P* < .05) were more common in the non-PWUD group. Both groups had a similar prevalence of septic arthritis (15.4% vs 10.2%; *P* = .30). All patients with isolated bacteremia had documented negative blood cultures prior to initiation of dalbavancin. Methicillin-sensitive *S aureus* (9% vs 18.4%; *P* = .12) and MRSA (15.4% vs 11.2%; *P* = .56) were the most common causative microorganisms in both PWUD and non-PWUD infections and there was no statistically significant difference between the 2 groups. Infections caused by streptococcal species and enterococcal species accounted for 7.4% and 3.4% of infections, respectively, with no statistically significant difference between PWUD and non-PWUD. About 16% (16.5%) of infections were polymicrobial and in 9.1% of infections, microbiology data were not identified.

**Table 2.** Infection Information and Microbiology

Variable	Total (n = 176)	PWUD (n = 78)	Non-PWUD (n = 98)	*P* Value
Infection type				
Isolated bacteremia	82 (46.6)	26 (33.3)	56 (57.1)	<.01
Endocarditis	21 (11.9)	16 (20.5)	5 (5.1)	<.01
Nonvertebral osteomyelitis	38 (21.6)	11 (14.1)	27 (27.6)	<.05
Epidural abscess	8 (4.6)	7 (9)	1 (1)	<.05
Vertebral osteomyelitis	7 (2.3)	7 (9)	0	<.01
Septic arthritis	22 (12.5)	12 (15.4)	10 (10.2)	.30
>1 infection type^a^	2 (1.1)	1 (1.3)	1 (1)	1.00
Microbiology				
MSSA only	25 (14.2)	7 (9)	18 (18.4)	.12
MRSA only	23 (13.1)	12 (15.4)	11 (11.2)	.56
Streptococcal species only	13 (7.4)	5 (6.4)	8 (8.2)	.88
Enterococcal species only	6 (3.4)	1 (1.3)	5 (5.1)	.33
Polymicrobial^b^	29 (16.5)	12 (15.4)	17 (17.3)	.89
No microbiology identified	16 (9.1)	6 (7.7)	10 (10.2)	.76

Data are presented as No. (%) unless otherwise indicated.

Abbreviations: MRSA, methicillin-resistant *Staphylococcus aureus*; MSSA, methicillin-sensitive *Staphylococcus aureus*; PWUD, people who use drugs.

^a^Excludes isolated bacteremia.

^b^Infections with >1 causative organism.

Hospitalization and treatment information is summarized in Table 3. Average hospital length of stay in the PWUD group was 11.2 days (standard deviation [SD], 9.8) versus 11.3 days (SD, 14) in the non-PWUD group (*P* = .96). Non-PWUD were more likely to have an IV catheter at discharge (5.1% vs 21.4%; *P* < .01). Two of 16 PWUD and 1 of 5 non-PWUD who had endocarditis underwent at least 1 of the following: valve surgery, catheter-based aspiration, or cardiac device removal. Six of 11 PWUD and 10 of 27 non-PWUD who had nonvertebral osteomyelitis underwent at least 1 debridement. Two of 7 PWUD and 0 of 1 non-PWUD who had an epidural abscess underwent at least 1 drainage. One of 7 PWUD who had vertebral osteomyelitis underwent at least 1 debridement. Eleven of 12 PWUD and 9 of 10 non-PWUD who had septic arthritis underwent at least 1 washout. PWUD were treated with antibiotics an average of 12.2 days (SD, 10.1) versus 9.4 days (SD, 9.6) in the non-PWUD group prior to initiation of dalbavancin (*P* = .13). A similar percentage of PWUD (84.6%) and non-PWUD (85.7%) completed the entire intended course of dalbavancin that was determined by infectious diseases consultants at the initiation of dalbavancin use (*P* = .27). Approximately 60% (60.3%) of PWUD versus 58.2% of non-PWUD received 1 dose of dalbavancin (*P* = .90), 25.6% of PWUD versus 30.1% of non-PWUD received 2 doses of dalbavancin (*P* = .58), and 15.4% of PWUD versus 12.2% of non-PWUD received >2 doses of dalbavancin (*P* = .70). A similar percentage of PWUD (46.2%) and non-PWUD (32.7%) were treated with an additional antibiotic in conjunction with dalbavancin (*P* = .09). Detailed information about each treatment regimen by infection type is summarized in Supplement 1.

**Table 3.** Hospitalization and Treatment Information

Variable	Total (n = 176)	PWUD (n = 78)	Non-PWUD (n = 98)	*P* Value
Length of hospital stay, d, average (SD)	11.3 (12.3)	11.2 (9.8)	11.3 (14)	.96
IV catheter at discharge	25 (14.2)	4 (5.1)	21 (21.4)	<.01
Days of antibiotic use prior to dalbavancin, mean (SD)	10.6 (9.9)	12.2 (10.1)	9.4 (9.6)	.13
Completed intended course of dalbavancin	150 (85.2)	66 (84.6)	84 (85.7)	.27
Completed 1 dalbavancin dose^a^	104 (59.1)	47 (60.3)	57 (58.2)	.90
Completed 2 dalbavancin doses^a^	50 (28.4)	20 (25.6)	30 (30.1)	.58
Completed >2 dalbavancin doses^a^	24 (13.6)	12 (15.4)	12 (12.2)	.70
Prescribed an additional antibiotic in conjunction with dalbavancin	68 (38.6)	36 (46.2)	32 (32.7)	.09

Data are presented as No. (%) unless otherwise indicated.

Abbreviations: IV, intravenous; PWUD, people who use drugs; SD, standard deviation.

^a^Detailed dosing information is shown in Supplement 1.

Outcomes are summarized in Table 4. Assuming loss to follow-up did not achieve clinical cure, 73.1% of PWUD and 74.5% of non-PWUD achieved clinical cure at 90 days (*P* = .08). More than three-quarters (77.3%) of PWUD who completed the intended course of dalbavancin achieved clinical cure, and 50% of PWUD who did not complete the intended course achieved clinical cure. Seventy-five percent of non-PWUD who completed the intended course of dalbavancin achieved clinical cure, and 71.4% of non-PWUD who did not complete the intended course achieved clinical cure. PWUD were more likely than non-PWUD to have a PDD (26.9% vs 3.1%; *P* < .001). Approximately two-thirds (65.6%) of PWUD who received MOUD had a PDD, and 29.7% of PWUD with a history of IV drug use in the past 2 weeks had a PDD. Additionally, PWUD were more likely than non-PWUD to be lost to follow-up (20.5% vs 7.14%; *P* < .01), and 14.3% of PWUD compared to 0 of non-PWUD who had a PDD were lost to follow-up (*P* = .17). Of the PWUD with a PDD, 71.4% achieved clinical cure at 90 days versus 66.7% of non-PWUD with a PDD (*P* < .001).

**Table 4.** Outcomes

Variable	Total (n = 176)	PWUD (n = 78)	Non-PWUD (n = 98)	*P* Value
Loss to follow-up	23 (13.1)	16 (20.5)	7 (7.14)	<.01
PDD	24 (13.6)	21 (26.9)	3 (3.1)	<.001
PDD and loss to follow-up	3 (12.5)	3 (14.3)	0	.17
Clinical cure at 90 d	130 (73.9)	57 (73.1)	73 (74.5)	.08
PDD and clinical cure at 90 d	17 (70.8)	15 (71.4)	2 (66.7)	<.001

Data are presented as No. (%) unless otherwise indicated.

Abbreviations: PDD, patient-directed discharge; PWUD, people who use drugs.

## DISCUSSION

This retrospective multisite study is the largest real-world study of dalbavancin use for invasive gram-positive infections in PWUD and 1 of the first to compare outcomes between PWUD and non-PWUD within the same healthcare system. In this study, there was a high rate of clinical cure at 90 days after dalbavancin use in both PWUD and non-PWUD, demonstrating strong efficacy of dalbavancin across different populations, social circumstances, and infection types. However, there was a higher rate of PDD and loss to follow-up among PWUD and, because we considered all patients lost to follow-up as clinical failure, the actual rate of clinical cure within the PWUD group may be an underestimate. Our findings are consistent with prior studies that have demonstrated success with dalbavancin for invasive gram-positive infections in PWUD, as well as high rates of losses to follow-up [14–17]. In Bryson-Cahn et al, of 32 PWUD, 56% achieved clinical cure at 1 year after dalbavancin use for an invasive *S aureus* infection with 31% lost to follow-up [14]. Our higher rate of PDD among PWUD is similarly consistent with other studies [18].

The consistently high rate of PDD among PWUD across studies is a key contextual factor to consider as PDD is a commonly cited rationale for dalbavancin use in clinical practice. In our study, PWUD with a PDD had a high rate of clinical cure of 71.4%. One potential explanation for why this is higher than in other reports is that in our study, 41% of patients with opioid use disorder were continued/initiated on MOUD during admission. MOUD is systematically underprescribed to patients who are hospitalized with infections related to drug use, with rates of MOUD initiation between 7.8% and 28% [19–21]. Several studies have shown the benefits of co-management of serious infections and substance use disorder [22–24]. The relatively high cure rate in this subgroup in our study highlights the importance of addressing upstream factors that drive engagement in care such as treatment of substance use disorder and addressing housing insecurity. While the rate of MOUD during admission in our study is higher than other similar studies, it leaves much room for improvement.

The high cure rate among PWUD undergoing PDD is particularly notable because there are several factors that would predict a lower cure rate in this population. When dalbavancin is used at the time of PDD, there is a chance that patients do not have source control, so this subpopulation may be at higher risk for clinical failure than patients who were optimally treated at the beginning of the infection. Furthermore, patients with PDD are at higher risk for 30-day readmission and higher rates of loss to follow-up, so clinical cure rates in retrospective studies are likely to be lower in this population irrespective of the choice of antibiotic [25]. In our study, there was a small number of patients who underwent source control, yet both groups had similar rates of clinical cure. The use of dalbavancin in the absence of source control warrants careful attention as cross-resistance to dalbavancin can emerge if infection persists during the months-long tail of subtherapeutic concentrations given its prolonged half-life [26].

There are several limitations to our study. First, it is a retrospective study at 3 large urban/suburban academic medical centers in a heavily physician-rich area with both infectious diseases and addiction expertise as well as ready access to outpatient infusion services, thereby limiting generalizability. Second, the study population was heterogeneous with regards to site and severity of infection, microbiology, and source control, so the results may be difficult to generalize to individual patients. Third, there were varied treatment regimens with respect to dose and number of dalbavancin administrations, duration and type of lead-in antibiotics, as well as duration and type of concurrent antibiotics if used, making it difficult to ascertain which aspects of a treatment regimen contributed most to clinical cure. Fourth, because the PWUD and non-PWUD groups are fundamentally different in their demographic characteristics and types of infection, it is impossible with the current study design to eliminate all possible confounding variables. These limitations highlight the need for dalbavancin to be studied in randomized controlled trials for each infection type to minimize confounding. If future observational data are used, a propensity-matched study design would be helpful to ascertain the true effect of dalbavancin within each population. Last, the large majority of the study population identified as non-Hispanic White and spoke English as their primary language. Future research is needed for patients who often face additional barriers to care [27], including patients of color and with non-English language preferences, to better understand systems designs that may optimize the use of dalbavancin in these particular patient populations.

Prospective randomized trials of dalbavancin for severe infections such as *S aureus* bacteremia are underway, and PWUD have been identified as a key subgroup [28]. Results from larger prospective trials will help to guide the use of long-acting lipoglycopeptides for severe gram-positive infections among different populations. While awaiting additional clinical data, the results of our study suggest dalbavancin can play an important role in treatment of patients with severe invasive gram-positive infections, and may be a particularly useful treatment option for people who have barriers to care, including drug use.

## Supplementary Data

Supplementary materials are available at *Open Forum Infectious Diseases* online. Consisting of data provided by the authors to benefit the reader, the posted materials are not copyedited and are the sole responsibility of the authors, so questions or comments should be addressed to the corresponding author.

## Supplementary Material

ofae186_Supplementary_Data

## Notes

***Author contributions.*** D. A. S.: Principal investigator, study design, data collection, and manuscript and data preparation. S. Z.: Co-investigator and study manager, study design, data collection, and manuscript and data preparation. M. L. P.: Study design, data collection, and manuscript preparation. J. S.: Data analysis. J. C. P. and B. D.: Study design and manuscript preparation. H. S.: Study design. J. M.: Study design and data collection. V. S.: Data collection and data analysis. K. F.-H., M. K., and S. P.: Data collection.

***Acknowledgments.*** We acknowledge and thank all those dedicated to providing excellent care to people who use drugs.

***Patient consent.*** The study was approved with waiver of consent by the Mass General Brigham Institutional Review Board.
